# Myogenetic Oligodeoxynucleotides as Anti-Nucleolin Aptamers Inhibit the Growth of Embryonal Rhabdomyosarcoma Cells

**DOI:** 10.3390/biomedicines10112691

**Published:** 2022-10-25

**Authors:** Naoki Nohira, Sayaka Shinji, Shunichi Nakamura, Yuma Nihashi, Takeshi Shimosato, Tomohide Takaya

**Affiliations:** 1 Department of Agricultural and Life Sciences, Faculty of Agriculture, Shinshu University, 8304 Minami-minowa, Kami-ina, Nagano 399-4598, Japan; 2 Department of Agriculture, Graduate School of Science and Technology, Shinshu University, 8304 Minami-minowa, Kami-ina, Nagano 399-4598, Japan; 3 Cellular and Molecular Biotechnology Research Institute, National Institute of Advanced Industrial Science and Technology, Central 5-41, 1-1-1 Higashi, Tsukuba 305-8565, Japan; 4 Department of Biomolecular Innovation, Institute for Biomedical Sciences, Shinshu University, 8304 Minami-minowa, Kami-ina, Nagano 399-4598, Japan

**Keywords:** aptamer, embryonal rhabdomyosarcoma, myogenetic oligodeoxynucleotide, nucleolin

## Abstract

Embryonal rhabdomyosarcoma (ERMS) is the muscle-derived tumor retaining myogenic ability. iSN04 and AS1411, which are myogenetic oligodeoxynucleotides (myoDNs) serving as anti-nucleolin aptamers, have been reported to inhibit the proliferation and induce the differentiation of myoblasts. The present study investigated the effects of iSN04 and AS1411 in vitro on the growth of multiple patient-derived ERMS cell lines, ERMS1, KYM1, and RD. RT-PCR and immunostaining revealed that nucleolin was abundantly expressed and localized in nucleoplasm and nucleoli in all ERMS cell lines, similar to myoblasts. Both iSN04 and AS1411 at final concentrations of 10–30 μM significantly decreased the number of all ERMS cells; however, their optimal conditions were different among the cell lines. In all ERMS cell lines, iSN04 at a final concentration of 10 μM markedly reduced the ratio of EdU^+^ cells, indicating the inhibition of cell proliferation. Quantitative RT-PCR or immunostaining of phosphorylated histone H3 and myosin heavy chain demonstrated that iSN04 suppressed the cell cycle and partially promoted myogenesis but did not induce apoptosis in ERMS cells. Finally, both iSN04 and AS1411 at final concentrations of 10–30 μM disrupted the formation and outgrowth of RD tumorspheres in three-dimensional culture mimicking in vivo tumorigenesis. In conclusion, ERMS cells expressed nucleolin, and their growth was inhibited by the anti-nucleolin aptamers, iSN04 and AS1411, which modulates several cell cycle-related and myogenic gene expression. The present study provides evidence that anti-nucleolin aptamers can be used as nucleic acid drugs for chemotherapy against ERMS.

## 1. Introduction

Rhabdomyosarcoma (RMS), occurring in striated muscles throughout the body, is the most frequent soft tissue tumor in children [1,2]. RMS is classified into embryonal, alveolar, pleomorphic, and spindle-cell/sclerosing subtypes. Embryonal RMS (ERMS) accounts 70% of childhood RMS and has a number of causative mutations, including Ras [3], Rb [1,4], and p53 [5]. These mutations in muscular lineages, such as mesenchymal stem cells, skeletal muscle stem cells (satellite cells), or myogenic precursor cells (myoblasts), cause ERMS to present impaired myogenic differentiation and hyper-activated proliferation [6,7,8]. Alveolar RMS has been known to associate with the chromosomal translocations on *PAX3* and *PAX7* genes encoding Pax3/7 myogenic transcription factors [1,2]. Spindle-cell/sclerosing RMS has *RAB3IP-HMGA2* fusion transcript and *FGFR4* mutation [9,10]. *FGFR4* gene is a direct target of Pax3 and serves as an intracellular inhibitor of FGF signaling to regulate myogenesis [11]. Furthermore, numerous muscle-specific microRNAs have been reported as biomarkers and therapeutic targets of RMS because of their oncogenic or tumor-suppressive actions for RMS progression [12]. Therefore, the agents modulating the disrupted myogenic program are anticipated to be novel effective drugs for RMS. The five-year survival rate of high-risk RMS patients is still <30% [13], which has not been improved by standard anti-tumor chemotherapy using actinomycin D, cyclophosphamide, ifosfamide, and vincristine [1,14]. In addition to pan-tumor drugs, RMS-targeted treatments need to be developed to update the therapeutic strategy.

Nucleic acid aptamers are single-strand short nucleotides that specifically bind to target molecules in a structure-dependent manner and are promising candidates for next-generation drugs against manifold diseases. Dozens of aptamers targeting tumoral proteins have been developed and their therapeutic effects on cancer cells have been studied [15]. We recently identified a series of 18-base myogenetic oligodeoxynucleotides (myoDNs) that facilitate the differentiation of myoblasts, while suppressing the cell growth [16,17,18,19]. One of the myoDNs, iSN04, serves as the aptamer directly binding to nucleolin that is a ubiquitous multifunctional phosphoprotein localized in the nucleus, cytoplasm, and plasma membrane, depending on the context of cellular processes [20]. In myoblasts, iSN04 antagonizes nuclear nucleolin to improve the p53 protein level translationally inhibited by nucleolin, and finally leads to myogenesis via activation of the p53 signaling pathway [16]. In cancers, nucleolin is frequently observed on the cell surface, which interacts with the ligands involved in proliferation and apoptosis [21]. For instance, the interaction of nucleolin with ErbB1 and Ras promotes proliferation [22], while that with Fas inhibits apoptosis [23]. Thus, several nucleolin inhibitors have been developed for application in cancer therapy. AS1411 is a 26-base guanine-rich anti-nucleolin aptamer that functions in the nucleus, cytoplasm, and plasma membrane. AS1411 has shown anti-cancer activity against acute myeloid leukemia in clinical trials [21,24]. The latest study reported that nucleolin is expressed with TBX3 in multiple sarcomas including RMS, and AS1411 exhibited anti-sarcoma activities with IC_50_ values of 5–18 μM [25]. In addition to its anti-tumor effect, our study demonstrated that AS1411 promotes myoblast differentiation to the same extent as iSN04 [16]. These findings suggest that the combined myogenetic and anti-carcinogenic abilities of anti-nucleolin aptamers might be beneficial for RMS therapy. The present study investigated whether iSN04 and AS1411 affect the growth and myogenesis of ERMS cells.

## 2. Materials and Methods

### 2.1. Oligodeoxynucleotides

Phosphorothioated iSN04 (5′-AGA TTA GGG TGA GGG TGA-3′) [16,17,18,19,26] was synthesized (GeneDesign, Osaka, Japan) and purified by reversed-phase high performance liquid chromatography (RP-HPLC) using XBridge BEH C18 column (pore size 130 Å; particle size, 2.5 μm; analytical size, 4.6 × 75 mm) (Waters, Milford, MA, USA) at 60 °C under ion-pairing condition to guarantee >95% of purity. AS1411 (5′-GGT GGT GGT GGT TGT GGT GGT GGT GG-3′), having a phosphodiester backbone, was synthesized and desalted (Integrated DNA Technologies, Coralville, IA, USA) [16,27]. iSN04 and AS1411 were dissolved in endotoxin-free water. An equal volume of endotoxin-free water, without iSN04 or AS1411, served as the negative control.

### 2.2. Two-Dimensional (2D) Cell Culture

Three ERMS cell line stocks were provided by JCRB Cell Bank (National Institutes of Biomedical Innovation, Health and Nutrition, Osaka, Japan): ERMS1 cells (JCRB1648) derived from the anaplastic pelvic ERMS of a 5-year-old female [5], KYM1 cells (JCRB0627) derived from the neck ERMS of a 9-month-old infant [28], and RD cells (JCRB9072) derived from the malignant pelvic ERMS of a 7-year-old female [29]. The ERMS cells were maintained in RPMI1640 (2 g/L glucose) (Nacalai, Osaka, Japan) with 10% fetal bovine serum (HyClone; GE Healthcare, Salt Lake City, UT, USA), 100 units/mL penicillin, and 100 μg/mL streptomycin at 37 °C with 5% CO_2_ [30]. The commercially available human myoblasts isolated from the healthy subject (CC-2580; Lonza, Walkersville, MD, USA) were maintained in Skeletal Muscle Growth Media-2 (CC-3245; Lonza) on the dishes coated with collagen type I-C (Cellmatrix; Nitta Gelatin, Osaka, Japan) at 37 °C with 5% CO_2_ [16,17,19,31].

### 2.3. Immunocytochemistry

The cells were fixed with 2% paraformaldehyde, permeabilized with 0.2% Triton X-100, and immunostained with 1.0 μg/mL rabbit polyclonal anti-nucleolin antibody (ab22758; Abcam, Cambridge, UK) [16,18], 1:1000 of rabbit monoclonal anti-phosphorylated histone (p-histone) H3 (Ser10) antibody (D2C8; Cell Signaling Technology, Danvers, MA, USA), or 0.5 μg/mL mouse monoclonal anti-pan-myosin heavy chain (MHC) antibody (MF20; R&D Systems, Minneapolis, MN, USA) [16,17,19,31]. A total of 0.1 μg/mL Alexa Fluor 488- or 594-conjugated donkey polyclonal anti-rabbit or -mouse IgG antibody (Jackson ImmunoResearch, West Grove, PA, USA) was used as a secondary antibody. Cell nuclei were stained with DAPI (Nacalai). Fluorescent images were captured using EVOS FL Auto microscope (AMAFD1000; Thermo Fisher Scientific, Waltham, MA, USA). The ratio of p-histone H3^+^ cells was defined as the number of p-histone H3^+^ nuclei divided by the total number of nuclei using Image J software version 1.52a (National Institutes of Health, Bethesda, MD, USA).

### 2.4. Cell Counting

Sets of 5.0 × 10^4^ cells/well were seeded on 12-well (ERMS1 and RD) or 24-well (KYM1) plates and then treated with 10 or 30 μM of iSN04 or AS1411 the next day. After 72 h (ERMS1 and KYM1) or 92 h (RD) of treatment, when the control group became confluent, the cells were completely dissociated by 0.25% trypsin (Fujifilm Wako Chemicals, Osaka, Japan) and subjected to cell counting using a hemocytometer [30].

### 2.5. EdU (5-Ethynyl-2′-deoxyuridine) Staining

Sets of 1.0 × 10^5^ cells (ERMS1 and RD) or 3.0 × 10^5^ cells (KYM1) were seeded on 30-mm dishes and treated with 10 μM iSN04 the next day. After 24 h (ERMS1 and KYM1) or 48 h (RD) of treatment, EdU was administered at a final concentration of 10 μM, and the cells were cultured for 3 h. EdU staining was performed using the Click-iT EdU Imaging Kit (Thermo Fisher Scientific), according to the manufacturer’s instruction. Cell nuclei were visualized by DAPI staining. The ratio of EdU^+^ cells was defined as the number of EdU^+^ nuclei divided by the total number of nuclei using ImageJ software [31].

### 2.6. Quantitative Real-Time RT-PCR (qPCR)

Sets of 1.5 × 10^5^ cells (RD), 2.0 × 10^5^ cells (ERMS1), or 3.0 × 10^5^ cells (KYM1) were seeded on 30-mm dishes and treated with 10 μM iSN04 the next day. After 72 h (ERMS1 and KYM1) or 96 h (RD) of treatment, the total RNA from the cells was isolated using NucleoSpin RNA Plus (Macherey-Nagel, Düren, Germany) and reverse transcribed using ReverTra Ace qPCR RT Master Mix (TOYOBO, Osaka, Japan). qPCR was performed using GoTaq qPCR Master Mix (Promega, Madison, WI, USA) with the StepOne Real-Time PCR System (Thermo Fisher Scientific). The amount of each transcript was normalized to that of *GAPDH*. The results are presented as fold-changes. The primer sequences are listed in Table 1.

### 2.7. Three-Dimensional (3D) Culture of RD Tumorspheres

RD cells were dissociated and suspended in 3D Tumorsphere Medium XF (PromoCell, Heidelberg, Germany). Drops (300 cells/30 μL) were placed on 24-well floating-culture plates (Sumitomo Bakelite, Tokyo, Japan). Subsequently, the plates were turned over for the hanging-drop culture. After 3 days, the plates were turned over again, and 300 μL/well of 3D Tumorsphere Medium XF with 10 or 30 μM of iSN04 or AS1411 was added to RD tumorspheres (defined as day 0). The spheres were maintained without medium exchange for 10 days. Bright-field images were taken using EVOS FL Auto microscope [30].

### 2.8. Statistical Analysis

Results are presented as the mean ± standard error. Statistical comparison between two groups was performed using unpaired two-tailed Student’s *t*-test and among multiple groups using Scheffe’s *F* test following one-way analysis of variance. Statistical significance was set at *p* < 0.05.

## 3. Results

### 3.1. Nucleolin Expression and Localization in ERMS Cells

**Figure 1:**
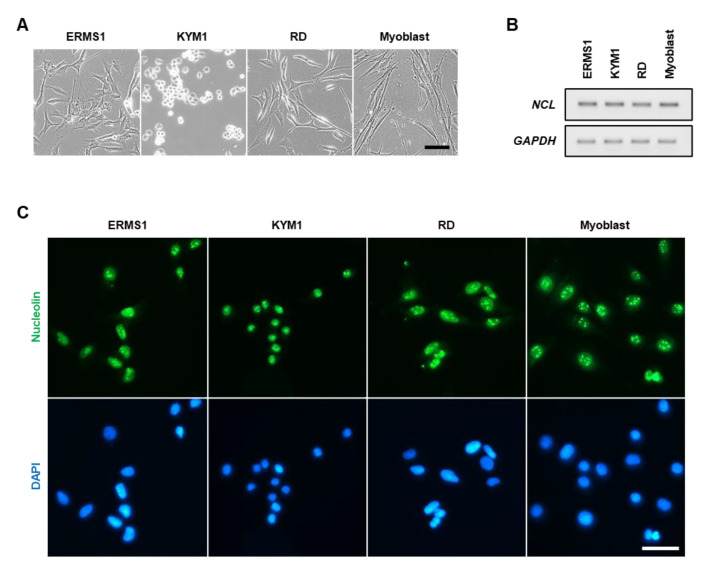
Expression and localization of nucleolin in ERMS cells. (**A**) Representative phase-contrast images of ERMS cells and myoblasts. Scale bar, 100 μm. (**B**) RT-PCR results of nucleolin (*NCL*) and *GAPDH* expression in ERMS cells and myoblasts. All the RT-PCR products were electrophoresed in one agarose gel and the image of each product was cropped to display. The image was not processed to adjust brightness, contrast, or any other properties. (**C**) Representative immunofluorescent images of nucleolin staining of ERMS cells and myoblasts. Scale bar, 50 μm.

Three patient-derived ERMS cell lines, ERMS1 [5], KYM1 [28], and RD [29], were used in this study. These cells are morphologically different from each other and from myoblasts, reflecting the diverse phenotypes of ERMS cells (Figure 1A). Initially, the expression and localization of nucleolin in these cells were confirmed. RT-PCR indicated that nucleolin (*NCL*) mRNA was abundantly transcribed in all ERMS cell lines as well as in myoblasts (Figure 1B). Immunostaining revealed that nucleolin localized in the nucleoplasm and intensively in the nucleoli but not in the cytoplasm and on the plasma membrane of all ERMS cells (Figure 1C). The localization patterns of nucleolin in these cells were similar to those observed in myoblasts.

### 3.2. iSN04 and AS1411 Inhibit the Growth of ERMS Cells

**Figure 2:**
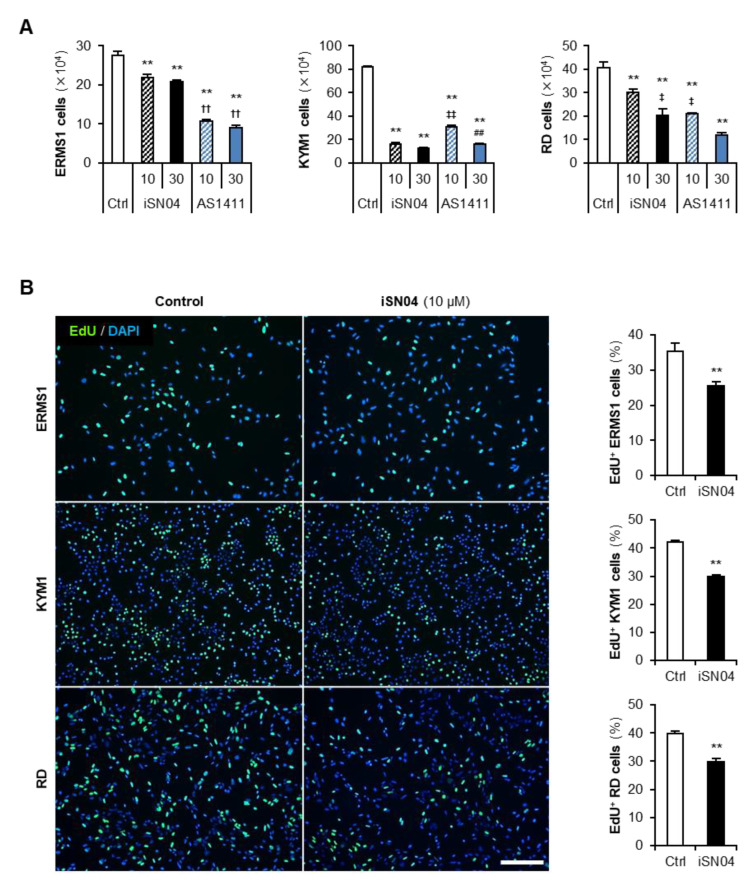
iSN04 and AS1411 inhibit the growth of ERMS cells. (**A**) Numbers of the ERMS cells treated with 10 or 30 μM of iSN04 or AS1411 for 72 h (ERMS1 and KYM1) or 96 h (RD). ** *p* < 0.01 vs. control; ^††^ *p* < 0.01 vs. 30 μM iSN04; ^‡^ *p* < 0.05, ^‡‡^ *p* < 0.01 vs. 10 μM iSN04; ^##^ *p* < 0.01 vs. 10 μM AS1411 (Scheffe’s *F* test). *n* = 4. (**B**) Representative images of the ERMS cells treated with 10 μM iSN04 for 24 h (ERMS1 and KYM1) or 48 h (RD). Scale bar, 200 μm. Ratio of EdU^+^ cells were quantified. ** *p* < 0.01 vs. control (Student’s *t*-test). *n* = 6.

Next, the effects of anti-nucleolin aptamers on the growth of ERMS cells were investigated. The cells were treated with iSN04 or AS1411 until their negative controls became confluent, then the number of cells was counted (Figure 2A). Both iSN04 and AS1411 significantly decreased the number of all ERMS cells compared to the control group. In ERMS1 cells, the inhibitory effects of both iSN04 and AS1411 were saturated at a concentration of 10 μM; however, the activity of AS1411 was markedly higher than that of iSN04. In contrast, KYM1 cells were more sensitive to iSN04 than to AS1411. In RD cells, 10 and 30 μM of iSN04 and AS1411 significantly reduced the cell numbers in a dose-dependent manner, and AS1411 showed higher activity compared to iSN04. These results demonstrate that anti-nucleolin aptamers are effective in inhibiting the growth of ERMS cells, but their activities or sensitivities to the cells may differ among cell lines.

To clarify the action of anti-nucleolin aptamers, ERMS cells treated with iSN04 were subjected to EdU staining, and the EdU^+^ cells replicating genomic DNA were quantified (Figure 2B). In all ERMS cell lines, iSN04 markedly decreased the ratio of EdU^+^ cells, indicating that iSN04 delayed the cell cycle. These results demonstrate that iSN04 suppresses cell proliferation, resulting in inhibitory effects on the growth of ERMS cell lines.

### 3.3. iSN04 Alters the Gene Expression in ERMS Cells

**Figure 3:**
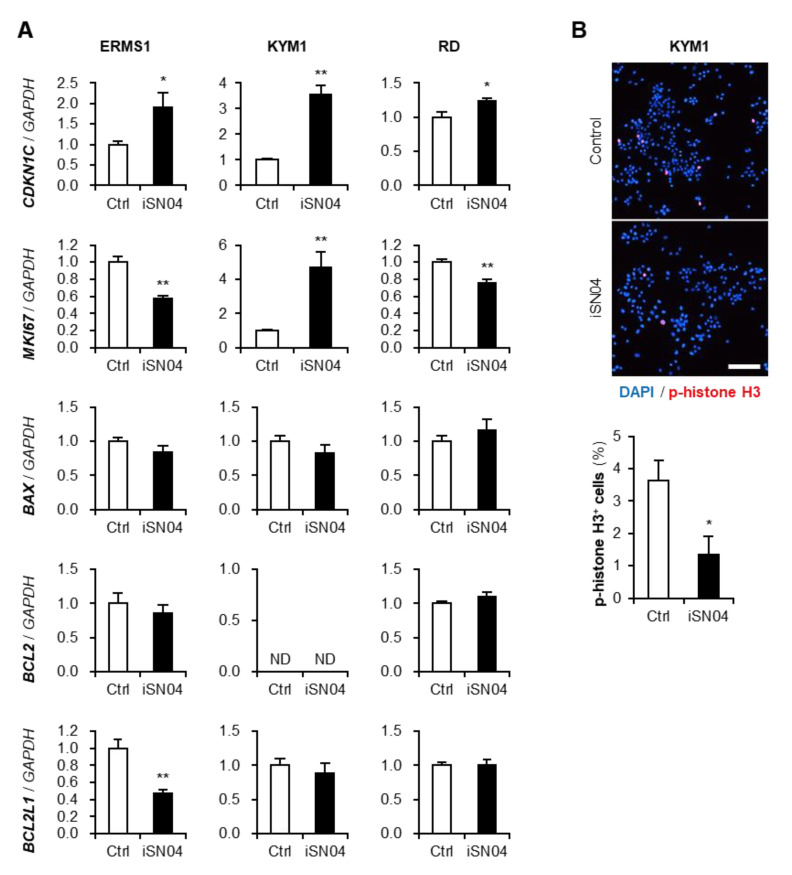
Effects of iSN04 on the proliferative and apoptotic gene expression in ERMS cells. (**A**) qPCR quantified the mRNA levels of p57^Kip2^ (*CDKN1C*), Ki-67 (*MKI67*), Bax (*BAX*), Bcl-2 (*BCL2*), and Bcl-xL (*BCL2L1*) in the ERMS cells treated with 10 μM iSN04 for 72 h (ERMS1 and KYM1) or 96 h (RD). Mean value of control group was set to 1.0 in each gene. ND, not detected. * *p* < 0.05, ** *p* < 0.01 vs. control (Student’s *t*-test) *n* = 4. (**B**) Representative immunofluorescent images of p-histone H3 staining of the KYM1 cells treated with 10 μM iSN04 for 48 h. Scale bar, 100 μm. Ratio of p-histone H3 cells were quantified. * *p* < 0.05 vs. control (Student’s *t*-test). *n* = 4.

The effects of iSN04 on the gene expression in ERMS cells were quantified by qPCR (Figure 3A). iSN04 significantly increased the mRNA levels of cyclin-dependent kinase inhibitor 1C (p57^Kip2^) (*CDKN1C*) in all ERMS cell lines, and decreased the transcription of proliferation marker Ki-67 (*MKI67*) in ERMS1 and RD cells, which corresponded well with the results of EdU staining. Although the *MKI67* mRNA level in KYM1 cells was unpredictably elevated by iSN04, immunostaining of phosphorylated histone (p-histone) H3, a marker of cell-cycle shift from G_2_ to M phase, visualized that iSN04 treatment significantly reduced the ratio p-histone H3^+^ cells (Figure 3B). The mRNA levels of apoptosis-related factors, Bax (*BAX*), Bcl-2 (*BCL2*), and Bcl-xL (*BCL2L1*), were not altered by iSN04 in every ERMS cell line, except for BCL2L1 in ERMS1 cells. These data demonstrate that iSN04 attenuates the growth of ERMS cells by inhibiting the cell cycle but not by inducing apoptosis.

**Figure 4:**
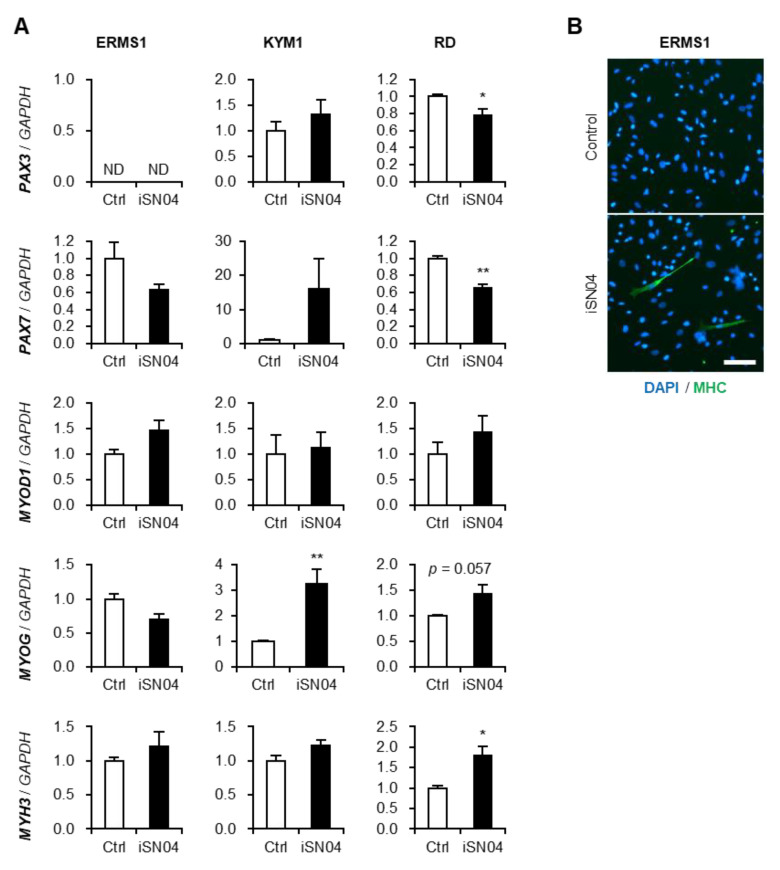
Effects of iSN04 on the myogenic gene expression in ERMS cells. (**A**) qPCR quantified the mRNA levels of Pax3 (*PAX3*), Pax7 (*PAX7*), MyoD (*MYOD1*), myogenin (*MYOG*), and embryonic MHC (*MYH3*) in the ERMS cells treated with 10 μM iSN04 for 72 h (ERMS1 and KYM1) or 96 h (RD). Mean value of control group was set to 1.0 in each gene. ND, not detected. * *p* < 0.05, ** *p* < 0.01 vs. control (Student’s *t*-test) *n* = 4. (**B**) Representative immunofluorescent images of MHC staining of the ERMS1 cells treated with 10 μM iSN04 for 48 h. Scale bar, 50 μm.

iSN04, as a myoDN, not only suppresses the proliferation but also promotes the myogenic differentiation of myoblasts [16,17,18,19]. Since ERMS cells retain myogenic abilities even though they are dysregulated [7], we investigated whether iSN04 upregulates the myogenic gene expression in ERMS cells (Figure 4A). In RD cells, iSN04 significantly decreased the mRNA levels of Pax3 (*PAX3*) and Pax7 (*PAX7*), which are undifferentiated myogenic transcription factors. Although iSN04 did not change the expression of MyoD (*MYOD1*) as a master regulator of myogenic program, it markedly induced myogenin (*MYOG*) as a myogenic transcription factor in KYM1 cells and embryonic myosin heavy chain (MHC) (*MYH3*) as a sarcomeric protein in RD cells. Although inductions of these myogenic genes were not detected by qPCR in ERMS1 cells, immunostaining of pan-MHC revealed that iSN04 leads transformation of ERMS1 cells into MHC^+^ myocyte-like cells (Figure 4B). Overall, iSN04 tended to induce myogenic differentiation, but its effects were partial and divergent among ERMS cell lines.

### 3.4. iSN04 and AS1411 Disturb the Formation of RD Tumorspheres

**Figure 5:**
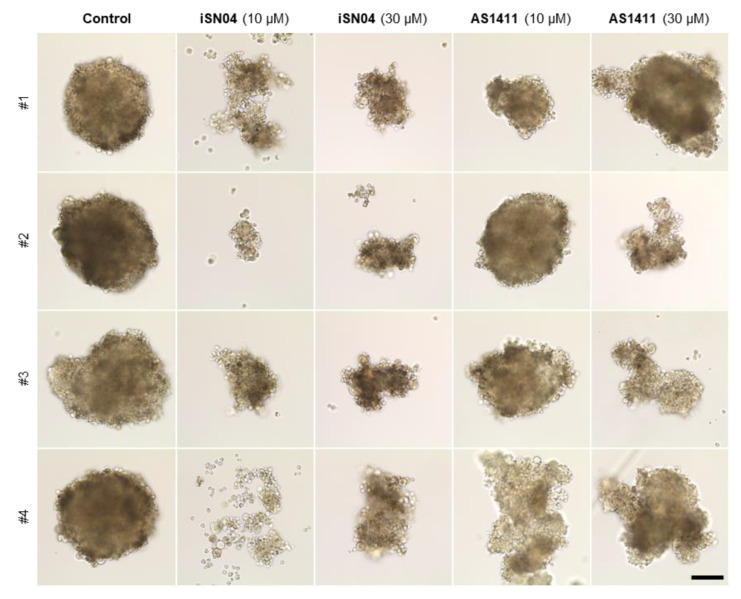
iSN04 and AS1411 disturb the formation of RD tumorspheres. Representative bright-field images of the RD tumorspheres treated with 10 or 30 μM of iSN04 or AS1411 for 10 days. Scale bar, 100 μm.

The 3D culture of tumorspheres is a valuable method to mimic in vivo tumorigenesis because the cells in spheres can exert inherent characteristics of cancer stem cells [33]. We have recently developed a xeno-free floating culture system for tumorspheres of RD cells [30]. The initial RD aggregations formed by hanging-drop culture were subsequently subjected to floating culture with iSN04 or AS1411 (Figure 5). In the control group, RD tumorspheres stably grew for 10 days and eventually shaped approximately 0.4 mm-diameter globes. However, iSN04- or AS1411-treatment disturbed the sphere formation. AS1411 interfered with the growth of some RD spheres, which indicated abnormal shapes and small diameters. iSN04 disrupted the formation and growth of RD tumorspheres more severely than AS1411. iSN04-treatment arrested the outgrowth of RD aggregation and broke some of them in small clusters. These results suggest that anti-nucleolin aptamers are effective in suppressing ERMS tumorigenesis, particularly in small metastatic lesions.

## 4. Discussion

The present study indicated that multiple ERMS cell lines express nucleolin, and their growth is inhibited by anti-nucleolin aptamers, iSN04 and AS1411. A number of cancer studies have provided evidence for the contribution of nucleolin in tumorigenesis. The localization and function of nucleolin varies in cancers [21]. Cell surface nucleolin is involved in ErbB1- and Ras-regulated proliferation [22], and it blocks Fas-induced apoptosis [23]. Cytoplasmic nucleolin binds to mRNAs of Bcl-2 and Bcl-xL to stabilize them, thereby protecting cancers against apoptosis [34,35]. However, in ERMS cells, nucleolin was not detected on the plasma membrane or in the cytoplasm. Correspondingly, antagonizing nucleolin by iSN04 did not alter the mRNA levels of Bcl-2 and Bcl-xL. In ERMS cells, nucleolin was localized in the nucleoli. Nucleolar nucleolin has been reported to interact with ribosomal DNA (rDNA) and increase the RNA polymerase I transcription [36]. This process is commonly activated in tumors because hyper-proliferative cancer cells require a large amount of protein synthesis. Therefore, nucleolar nucleolin is considered to play an indispensable role in the growth of cancer cells [21]. This could be one of the mechanisms by which anti-nucleolin aptamers attenuate the growth of ERMS cells. Both iSN04 and AS1411 are speculated to interact with the RNA-binding domains of nucleolin [16,18,24]. These aptamers might trap nucleolar nucleolin by competing with rDNA in ERMS cells. Several targets of nucleolin in sarcoma have been identified. In osteosarcoma, nucleolin is involved in GSK3β-mediated stability of HIF1α mRNA [37]. The latest study reported wide expression of nucleolin in fibrosarcoma, chondrosarcoma, liposarcoma, synovial sarcoma, and RMS. In these sarcomas, nucleolin is co-expressed with TBX3 to regulate p21^CIP1^ and p14^ARF^ transcriptions [25]. Other studies indicated that colony stimulating factor 1 (CSF-1) is overexpressed in solid tumors to recruit blood cells to suppress tumor immunity with changing their microenvironment [38,39]. An inhibitor of CSF-1 receptor impaired metastasis of RMS in mice [39]. Intriguingly, nucleolin binds to CSF-1 mRNA to promote its translation in breast cancer cells [40,41]. These findings demonstrate that nucleolin and its targets are potentially involved in many processes of development, growth, and metastasis of RMS. The action mechanisms of anti-nucleolin aptamers together with the role of nucleolin in RMS should be further elucidated.

In addition to that, some limitations of iSN04 and AS1411 need to be overcome for clinical application in the future, mainly because of ubiquitous expression of nucleolin. Although iSN04 did not alter apoptotic gene expression in ERMS cells, cytotoxicity of iSN04 and AS1411 both in various RMS subtypes and normal cells is necessary to be assessed by MTT assay, for example [42]. From the point of view of physiological condition, glucose addiction is a popular characteristics of tumors. A recent study reported that glucose availability is required for aggressiveness of RD cells [43]. In myoblasts, we confirmed that iSN04 can improve the impaired myogenic differentiation by excessive glucose as a model of diabetes mellitus [17]. Examination of anti-RMS effects of iSN04 and AS1411 in a high glucose concentration would provide useful information for in vivo experiments. Finally, the drug delivery system carrying anti-nucleolin aptamers to RMS is required to be developed. The proteomics analysis comparing RMS and normal muscle identified dozens of cell surface proteins specifically expressed in RMS [44]. They would be biomarkers and might be available as hooks to incorporate anti-nucleolin aptamers specifically into RMS cells.

In general, aptamers are set to target the membrane or extracellular proteins in view of drug action and are developed by the systemic evolution of ligands by exponential enrichment (SELEX) [45]. In contrast, iSN04 was identified as a myoDN that promotes myogenic differentiation [16], and AS1411 was discovered as an anti-proliferative nucleotide [24]. Nuclear nucleolin was defined as their target a posteriori. Both 18-base iSN04 and 26-base AS1411 have been confirmed to be incorporated into the cytoplasm without any carriers or transfection reagents [16,24], probably because of their short sequences compared to the average length of the known aptamers (51 bases) [46]. Single-strand short DNA is usually taken up by cells through the endocytic process termed gymnosis [47], which enables compact DNA aptamers to target cytoplasmic and nuclear proteins. In addition to the anti-cancer aptamers working outside the cells [15], iSN04 and AS1411 present alternative directions for the development of therapeutic aptamers targeting the intracellular factors. It should be noted that working concentrations of iSN04 and AS1411 (at final concentrations of 10–30 μM) were relatively high compared to those of small molecules, probably because only a part of them was taken up into cytoplasm. To improve cell permeabilities of anti-nucleolin aptamers, drug delivery systems, such as lipid nanoparticles, need to be tested. Turnover of iSN04 inside the cells has not yet been clarified. Since iSN04 is the phosphorothioated DNA resistant to nucleases, it might escape degradation and remain to accumulate in ERMS cells. In 2D culture for 4 days, iSN04 inhibited the growth of RD cells more severely at a concentration of 30 μM rather than 10 μM, while in 3D culture for 10 days, the effects of iSN04 were not markedly different between concentrations of 10 and 30 μM. As discussed above, iSN04 in the medium might be continuously incorporated and cumulatively function for several days due to its low permeability and high stability. It would be the reason that 10 μM iSN04 was sufficiently effective in tumorspheres.

The inhibitory activities of iSN04 and AS1411 on proliferation were different among the ERMS cell lines. This provides an important insight into the development of custom aptamers for tailor-made medicines. The previous studies suggested that AS1411 recognizes only a small subset of the total nucleolin [24,48], because nucleolin is post-translationally modified and forms a complex with various partners. Certain forms of nucleolin interplaying iSN04 and AS1411 might differ, which would impact the drug efficacy. This is also related to the myogenetic activity of iSN04 in ERMS cells. We recently demonstrated that both iSN04 and AS1411 robustly facilitate the myogenic differentiation of myoblasts [16]. However, induction of myogenic gene expression by iSN04 was partial and varied among the ERMS cell lines. In addition to the dysregulated myogenic program in ERMS cells, the presence of nucleolin might be distinct from that in myoblasts. To overcome these issues, it is necessary to enhance the myogenetic activity of anti-nucleolin aptamers in ERMS cells. For example, a histone deacetylase inhibitor (HDACI), trichostatin A, promotes myoblast differentiation by upregulating myogenic gene transcription [49]. Another HDACI, PXD-101, has been reported to induce myogenesis in RD cells by increasing MyoD, myogenin, and MHC expression [50]. Epigenetic alteration by HDACIs has the potential to synergistically improve the myogenetic activity of anti-nucleolin aptamers. The current standard chemotherapy for RMS is the combination treatment of multiple anti-tumor drugs [1,14]. For clinical application, the co-effects of anti-nucleolin aptamers with existing agents need to be validated in the future.

## 5. Conclusions

A putative sarcoma-related protein, nucleolin is localized in the nucleoli of multiple ERMS cell lines: ERMS1, KYM1, and RD. Regardless, without any carriers, two anti-nucleolin aptamers, iSN04 and AS1411, suppressed the proliferation of ERMS cells. By modulating gene expression, iSN04 impaired cell cycle and promoted myogenic differentiation but did not induce apoptosis in ERMS cells. Both iSN04 and AS1411 also inhibited the formation of RD tumorspheres in the 3D culture system. These results indicate that anti-nucleolin aptamers can be used as alternatives or potential drug candidates for chemotherapy to suppress ERMS tumorigenesis.

## 6. Patents

Shinshu University has been assigned the invention of iSN04 by T.T., Koji Umezawa, and T.S., and Japan Patent Application 2018-568609 was filed on 15 February 2018.

## Figures and Tables

**Table 1:** Primer Sequences for qPCR.

Gene	Sequence (5′-3′)	Reference
*BAX*	GCTGGACATTGGACTTCCTC CTCAGCCCATCTTCTTCCAG	[30]
*BCL2*	AACATCGCCCTGTGGATGAC GGCCGTACAGTTCCACAAAG	[32]
*BCL2L1*	GGCCACTTACCTGAATGACC AAGAGTGAGCCCAGCAGAAC	[30]
*CDKN1C*	GGCCTCTGATCTCCGATTTCTTC GGGTCTGCTCCACCGAG	[30]
*GAPDH*	TGTCAAGCTCATTTCCTGGTA GTGAGGGTCTCTCTCTTCCTCTTGT	[30]
*MKI67*	AAGAGGTGTGCAGAAAATCCAAAG CTTCACTGTCCCTATGACTTCTGGTT	[30]
*MYH3*	GGACAGGAAGAATGTGCTGAGATT GCCTCTTGTAGGACTTGACTTTCAC	[16]
*MYOD1*	TGCTCCGACGGCATGATGGAC TCGACACCGCCGCACTCT	[16]
*MYOG*	AACCCAGGGGATCATCTGCTCAC GTTGGGCATGGTTTCATCTGGGAAG	[16]
*NCL*	ATTGGTAGCAACTCCTGGTAAG CACTGTCATCATCCTCCTCTTC	[16]
*PAX3*	AGGAAGGAGGCAGAGGAAAG CAGCTGTTCTGCTGTGAAGG	[17]
*PAX7*	GACCCCTGCCTAACCACATC GTCTCCTGGTAGCGGCAAAG	[16]

## Data Availability

The raw data supporting the conclusions of this article will be made available by the authors, without undue reservation.
